# Supplementary material for: The impact of matches and travel on rugby players’ sleep, wellness and training
Source: PLoS One. 2022 Feb 8;17(2):e0261517. doi: 10.1371/journal.pone.0261517 (PMC8824341; doi:10.1371/journal.pone.0261517)
Supplement: S1 Table — (DOCX) [file pone.0261517.s001.docx]

| **Supplementary Table 1: Mediating effects of sleep on wellness for each monitored team, with ±90% compatibility limits. All units are minutes.** | | |
| --- | --- | --- |
| **Team** | **Between-players effect** | **Within-players effect** |
| A | **-3.6, ±3.8 T*^0^** | **-0.2, ±1.8 T****** |
| B | **11.2, ±7.1 S**** | **1.1, ±1.7 T****** |
| C | 3.6, ±4.9 T*^0^ | **10.0, ±1.3 S****** |
| D | **-7.5, ±5.1 S**** | 1.5, ±1.4 T*** |
| Observed magnitude: T, trivial; S, small; M, moderate; L, large.  Reference-Bayesian likelihoods of true substantial change: *possibly; **likely; ***very likely, ****most likely; *** and **** indicate rejection (p <0.05 and <0.005 respectively) of the non-superiority or non-inferiority hypothesis.  Reference-Bayesian likelihoods of true trivial change: ^0^possibly; ^00^likely; ^000^very likely, ^0000^most likely.  Effects in **bold** have adequate precision at the 99% level (rejection of the superiority or inferiority hypothesis, p<0.005). | | |
