# Supplementary material for: The impact of matches and travel on rugby players’ sleep, wellness and training
Source: PLoS One. 2022 Feb 8;17(2):e0261517. doi: 10.1371/journal.pone.0261517 (PMC8824341; doi:10.1371/journal.pone.0261517)
Supplement: S2 Table — (DOCX) [file pone.0261517.s002.docx]

| **Supplementary Table 2. Changes in Total Distance (TD, metres), Total Distance at High-Speed (TDHS, metres), Total Relative Distance (TRD, metres/minutes) and Internal Load (IL, Arbitrary Units) for training overseas and upon return.** | | | | | | | | |
| --- | --- | --- | --- | --- | --- | --- | --- | --- |
| **Team** | **TD changes, ±90%CL** | | **TDHS changes, ±90%CL** | | **TRD changes, ±90%CL** | | **IL changes, ±90%CL** | |
|  | **Overseas** | **Return** | **Overseas** | **Return** | **Overseas** | **Return** | **Overseas** | **Return** |
| A | **3086, ±723 XL****** | 131, ±311 T** | **265, ±125 L****** | **-102, ±68 S**** | **-5.1, ±4.2 S**** | **-9.1, ±3.3 M****** | n/a | n/a |
| B | -29, ±335 T | -116, ±333 T** | n/a | n/a | 1.3, ±3.3 T^0^* | **-2.9, ±2.5 S*** | **-289, ±51 M****** | **-36, ±48 T^0^*** |
| C | n/a | n/a | n/a | n/a | n/a | n/a | **-74, ±42 S**** | **-19, ±44 T**** |
| D | **-566, ±316 S**** | n/a | **-566, ±316 S**** | n/a | **-18.8, ±2.8 L****** | n/a | **-201, ±78 M*^0^** | n/a |
| CL, compatibility limits.  Observed magnitude: T, trivial; S, small; M, moderate; L, large.  Reference-Bayesian likelihoods of true substantial change: *possibly; **likely; ***very likely, ****most likely; *** and **** indicate rejection (p <0.05 and <0.005 respectively) of the non-superiority or non-inferiority hypothesis.  Reference-Bayesian likelihoods of true trivial change: ^0^possibly; ^00^likely; ^000^very likely, ^0000^most likely.  Effects in **bold** have adequate precision at the 99% level (rejection of the superiority or inferiority hypothesis, p<0.005).  Effects with CL but without likelihoods have inadequate precision.  n/a indicates data were not available. | | | | | | | | |
