# Supplementary material for: The impact of matches and travel on rugby players’ sleep, wellness and training
Source: PLoS One. 2022 Feb 8;17(2):e0261517. doi: 10.1371/journal.pone.0261517 (PMC8824341; doi:10.1371/journal.pone.0261517)
Supplement: Attachment — Submitted filename: Response to Reviewers.docx [file pone.0261517.s003.docx]

Thank you for the feedback on our manuscript. Your suggestions will help increase the quality of our work. We modified the manuscript based on your recommendations when possible. Please see below our answers to your specific comments.

**p5, Table 1: the number of time zones crossed is informative but it would also help to provide the reader with context if this table contained data on flight durations and distance.**

As professional rugby clubs were involved in the study, we had to limit the information provided to avoid teams’ identification. A reader provided with all travel information could easily identify the participating teams. Given the number of time zones crossed and direction of travel are enough to determine the effect of jet lag, or lack thereof, we prefer to exclude all other data to maintain confidentiality.

**p5, L86: as the authors are aware, the software associated with the activity monitors used in this study can determine a range of variables in addition to sleep duration (e.g. sleep onset latency, sleep efficiency % etc.). Whilst duration should clearly be included, I feel like much more insight could be gained by the analysis and inclusion of extra variables. Furthermore, the reporting of variables from the sleep diary such as bed time and wake up time could be obvious additions. This is probably the major limitation of the work but if the data exists, it would substantially enhance the manuscript.**

We focused on what was, in our opinion, the most important sleep variable, which is sleep duration. Furthermore, the analyses we performed included already multiple effects. Including further sleep variables would have reduce the precision of our analyses, increased the inflation of error that arises from analysing multiple effects and the risk to misinterpret effects that occurred by chance as substantial. Furthermore, given that our participants were professional athletes monitored in competition, some of the sleep diaries were incomplete and therefore, the data were not included. We acknowledged the exclusion of the other sleep variables on the limitation paragraph, but we rephrased our document to make our justification of the exclusion more clear (Line 416-418, reviewed manuscript).

**p6 L96: The specifics of the individual team wellness scales would benefit from further explanation. Given the "items differed in number and wording", I'm not completely clear on whether they are essentially considering the same wellness components (e.g. soreness, fatigue etc. etc.) or something different (which obviously impacts the interpretation of the impact of travel/sleep on wellness). Whilst many researchers (me included) have published work involving customised wellness questionnaires, the lack of validation of these inventories should be acknowledged. They may well prove to have validity similar to established tools such as the DALDA, MTDS, ARSS/SRSS, but until then we should probably be cautious with any findings.**

We agree that we did not made clear that the scales were different and, although some items where in common, other were exclusive to a specific scale. We rephrased our sentence to clarify these differences (Line 96-98, reviewed manuscript).

**p6, L103: it might be worth checking the specifications of the SPI HPU units. As far as I know, they use a GPS chip that samples at 10Hz and this data is supplemented with information from the accelerometers. Their suggestion that the device samples at 15Hz might not be technically correct.**

We checked the sample rate of the SPI HPU units and corrected it to 10Hz (Line 104, reviewed manuscript).

**p8, L154: see previous comments regarding wellness.**

As all scales were different, as explained in the methods, all responses were rescaled linearly to a score ranging from 0 to 100 to overcome such differences. Even if we agree with this reviewer that the differences in the scales may impact the interpretation of our results, which we acknowledged in the manuscript and this rebuttal, the choice of our smallest important was a compromise between a standardised effect and the standardised effect of a psychosomatic scale ranging from 0 to 100.

**p11, Table 2 (and all subsequent tables): whilst I completely understand the challenge in presenting statistical analysis results, the tables are potentially a little "busy". Trying to capture multiple levels of analysis with various symbols and formatting makes for somewhat confusing reading in the first instance. For example, "0" and "*" are each used for specific purposes. However, I acknowledge that after a few reads, the tables makes sense.**

**On a more general statistical analysis front, whilst I see the merit in levels of likelihoods of a true substantial change, this type of approach has been heavily criticised. My understanding is that the suggestion has been to either conduct a fully Bayesian analysis or a one-sided hypothesis test as an alternative (as has been done here). I'm aware that the authors are confident in suggesting that the method used is "Reference-Bayesian" but it should be acknowledged that not everyone seems to support this and I'm not aware of this journal's policy on this issue.**

We described and presented hypothesis testing results along with reference Bayesian analyses results to provide an assessment of sampling error from a frequentist and Bayesian perspective and give readers the opportunity to use their favourite perspective. As such, the table may appear busy on first read, but, as this reviewer kindly acknowledged, the tables make sense when carefully read. A possible problem with the reference-Bayesian analysis is justification of the non-informative prior, which we have now provided (negligible shrinkage with a weakly informative prior, and lack of bias with a non-informative prior) (Line 194-201, reviewed manuscript).

**p16, L315: it would be beneficial for the authors to provide some suggestions as to how the players who sleep more than average should take more care about avoiding sleep loss. What strategies could they employ?**

We agree that including sleeping strategies may be useful to readers. As all strategies to improve sleep can be used by all athletes, not only the one that sleep more than average, we included the main strategies that can be used to avoid sleep loss in the discussion (Line 333-335, reviewed manuscript).

**p17: as mentioned earlier, the discussion relating to the wellness results would benefit from being considered within the context of the potential limitations of the measurement tool.**

We agree with this reviewer, and we included the wellness scale issues in the limitations section of our manuscript (Line 402-410, reviewed manuscript).

**p18, L358: the sentence beginning "Athletes who feel well............" is a little to certain in my view. Consider rewriting to acknowledge the uncertainty about these links.**

We rewrote the sentence to acknowledge the uncertainty in the links between athletes feeling well and their performance and risk of injury (Line 378, reviewed manuscript).

**p18, L367: is it possible that an inherent lack of validity in the wellness scale is at least partly responsible for this lack of relationship?**

We agree with the reviewer comment, and we included an inherent lack of validity as a possible explanation for the lack of relationship between sleep and wellness in our results (Line 388-389, reviewed manuscript).

**p19, L390: this sentence relates to my previous point about additional sleep variables but I'm not completely sure what's meant here. Numerous variables can be obtained from the activity monitors**

As mentioned in our answer to a similar comment, we rephrased the manuscript to clarify the reason behind our decision to exclude all other sleep variables (Line 416-418, reviewed manuscript).

**p19: the inclusion of a limitations section is a strength and I believe that even with these limitations acknowledged that real world data on multiple teams is worth publishing. A general comment relating to the discussion section is that it parts it feels a tiny bit superficial. I know there is the limitation of word count, but there might be merit in more detailed discussion about the implications of the findings (I accept this can be limited due to the largely observational nature of the study) and perhaps some more information on possible strategies to mitigate the negative effects of this type of travel schedule and resultant changes to sleep.**

As acknowledged by this reviewer, the discussion points were limited by the observational nature of the study. However, as suggested by this reviewer, we included some implications of our findings, including the use of mitigating strategies, at the end of the manuscript discussion (Line 423-432, reviewed manuscript).

**OTHER**

On reflection, we noticed that we misreported some magnitude in Table 3 and we corrected them.
