# Supplementary material for: The impact of matches and travel on rugby players’ sleep, wellness and training
Source: PLoS One. 2022 Feb 8;17(2):e0261517. doi: 10.1371/journal.pone.0261517 (PMC8824341; doi:10.1371/journal.pone.0261517)
Supplement: Attachment — Submitted filename: Response to Reviewers.docx [file pone.0261517.s004.docx]

Thank you for the feedback on our manuscript. Your suggestions will help increase the quality of our work. We modified the manuscript based on your recommendations when possible. Please see below our answers to your specific comments.

**Please add some values to the results section. Even though you have included the statistical results in the tables I think its a bit easier to follow if you include eg effect sizes or means when you talk about small or moderate changes.**

One of the rules of scientific writing is not to repeat data in text and tables/figures. As it is, we have already repeated the qualitative magnitudes in the text to improve readability. With respect, we therefore have not shown repeated data, but to point the reader to the tables to find the data, we have added “as shown…” following mention of each table (Line 214-216, Line 223, Line 256 and Line 263-264, reviewed manuscript).

**Why have you analysed the teams separately instead of including a dummy variable per team to the model?**

As professional rugby clubs were involved in the study and data collection occurred across the Super Rugby competition, it was impossible to collect data in a standardised fashion. For example, the number of trips undertaken and the number of monitored days were not consistent between teams. Furthermore, each team players were monitored for only a relatively small number of nights. The heterogeneity of the data collected precluded us from run analyses that included all participant teams. We rephrased our document to clarify the reason behind our decision to run separate analyses for each team (Line 123-127, reviewed manuscript).

**OTHER**

On reflection, we noticed that we did not include a unit of measure in Table 4. Units of measure are now reported in all tables.
